# Ferroptosis Enhanced Diabetic Renal Tubular Injury *via* HIF-1α/HO-1 Pathway in db/db Mice

**DOI:** 10.3389/fendo.2021.626390

**Published:** 2021-02-18

**Authors:** Xiaomeng Feng, Shuo Wang, Zhencheng Sun, Hengbei Dong, Haitian Yu, Mengxiu Huang, Xia Gao

**Affiliations:** ^1^ Department of Endocrinology, Beijing Chao-Yang Hospital, Capital Medical University, Beijing, China; ^2^ Department of Infectious Diseases, Beijing Traditional Chinese Medical Hospital, Capital Medical University, Beijing, China; ^3^ Department of Osteology, Beijing Chao-Yang Hospital, Capital Medical University, Beijing, China; ^4^ Department of Reproductive Medicine, Beijing Obstetrics and Gynecology Hospital, Capital Medical University, Beijing, China; ^5^ Education Division, Beijing Chao-Yang Hospital, Capital Medical University, Beijing, China; ^6^ Department of Hepatobiliary, Beijing Chao-Yang Hospital, Capital Medical University, Beijing, China

**Keywords:** ferroptosis, diabetic nephropathy, renal tubular injury, heme oxygenase-1, hypoxia-inducible factor-1α

## Abstract

**Background:**

Ferroptosis is a recently identified iron-dependent form of cell death as a result of increased reactive oxygen species (ROS) and lipid peroxidation. In this study, we investigated whether ferroptosis aggravated diabetic nephropathy (DN) and damaged renal tubules through hypoxia-inducible factor (HIF)-1α/heme oxygenase (HO)-1 pathway in db/db mice.

**Methods:**

Db/db mice were administered with or without ferroptosis inhibitor Ferrostatin-1 treatment, and were compared with db/m mice.

**Results:**

Db/db mice showed higher urinary albumin-to-creatinine ratio (UACR) than db/m mice, and Ferrostatin-1 reduced UACR in db/db mice. Db/db mice presented higher kidney injury molecular-1 and neutrophil gelatinase-associated lipocalin in kidneys and urine compared to db/m mice, with renal tubular basement membranes folding and faulting. However, these changes were ameliorated in db/db mice after Ferrostatin-1 treatment. Fibrosis area and collagen I were promoted in db/db mouse kidneys as compared to db/m mouse kidneys, which was alleviated by Ferrostatin-1 in db/db mouse kidneys. HIF-1α and HO-1 were increased in db/db mouse kidneys compared with db/m mouse kidneys, and Ferrostatin-1 decreased HIF-1α and HO-1 in db/db mouse kidneys. Iron content was elevated in db/db mouse renal tubules compared with db/m mouse renal tubules, and was relieved in renal tubules of db/db mice after Ferrostatin-1 treatment. Ferritin was increased in db/db mouse kidneys compared with db/m mouse kidneys, but Ferrostatin-1 reduced ferritin in kidneys of db/db mice. Diabetes accelerated nicotinamide adenine dinucleotide phosphate (NADPH) oxidase-derived ROS formation in mouse kidneys, but Ferrostatin-1 prevented ROS formation derived by NADPH oxidases in db/db mouse kidneys. The increased malondialdehyde (MDA) and the decreased superoxide dismutase (SOD), catalase (CAT), glutathione peroxidases (GSH-Px) were detected in db/db mouse kidneys compared to db/m mouse kidneys, whereas Ferrostatin-1 suppressed MDA and elevated SOD, CAT, and GSH-Px in db/db mouse kidneys. Glutathione peroxidase 4 was lower in db/db mouse kidneys than db/m mouse kidneys, and was exacerbated by Ferrostatin-1 in kidneys of db/db mice.

**Conclusions:**

Our study indicated that ferroptosis might enhance DN and damage renal tubules in diabetic models through HIF-1α/HO-1 pathway.

## Introduction

Ferroptosis is a recently identified iron-dependent cell death, which is characterized by the increase of reactive oxygen species (ROS) to lethal levels (1). Iron is required for various vital processes such as heme synthesis, iron-sulfur cluster synthesis, and deoxyribonucleic acid synthesis (2). Iron also plays a critical role in the active sites of numerous enzymes which participate in the formation of nicotinamide adenine dinucleotide phosphate (NADPH) oxidases, xanthine oxidase, lysyl oxidase, and mitochondrial complex I and III (3).

However, the excess of iron in the cell damages cellular functions by producing ROS and ultimately leads to cell death (4). The formation of intracellular ROS is mainly through NADPH oxidases (5), which is regulated by iron (3). Lipid peroxidation is the damage by ROS on polyunsaturated fatty acids in cellular membranes or organelle membranes (6). Ferroptosis has been documented to be induced by lipid peroxidation which is caused by iron overloading (1). Iron-dependent lipid peroxidation is the oxidative process which is regulated by enzymatic antioxidants, such as superoxide dismutase (SOD), catalase (CAT), and glutathione peroxidases (GSH-Px) (6). GSH-Px includes multiple isoenzymes with different subcellular locations presenting distinct tissue-specific expression patterns (7). Glutathione peroxidase 4 (GPX4) is a specific and important regulator of ferroptotic cell death, since GPX4 can inhibit ferroptosis by repression of phospholipid peroxidation (8).

Heme is a main source of iron that synthesized (9). Heme oxygenase (HO)-1 is a phase II enzyme that metabolizes heme into biliverdin/bilirubin, carbon monoxide, and ferrous iron (10). HO-1 can be induced by a wide spectrum of cues, including inflammatory mediators, oxidants, and physical or chemical stimuli (10). Recent studies have suggested that HO-1 has a dual role in ferroptosis. The increasing studies have demonstrated that HO-1 acts as a key mediator in the cause of ferroptosis and plays a causative role for the development of several diseases (11–13), although there have been some researches indicating that HO-1 has protective effects against oxidative stress-related disorders (14). Hypoxia-inducible factor (HIF) is a heterodimer composed of a constitutive β-subunit and one of at least two different oxygen-dependent α-subunits (HIF-1α and -2α). The activity of HIF is mainly regulated by oxygen-dependent proteolysis of the α-subunits (15). HO-1 is one of the HIF target genes (15). Therefore, HIF-1α also regulates ferroptosis and is associated with the expression of GPX4 (16).

Diabetic nephropathy (DN) is the leading cause of end-stage renal disease. The previous studies of DN have mainly focused on glomeruli. However, recent data have shown that defects in tubules also result in albuminuria or proteinuria (17). Clinical observations in patients with type 1 diabetes highlighted the early involvement of the tubules in generating albuminuria (18). Subsequently, diabetic rats showed the decreased reabsorption of albumin in proximal tubules compared with the controls, despite of no promotion in glomerular filtration rate in diabetic rats (19). Additionally, no significant difference in glomerular sieving coefficient between diabetic rats and the controls was observed, while albuminuria presented in the diabetic rats (20). A good correlation has been found between urinary albumin excretion and the markers of tubular dysfunction. All these researches suggested that albuminuria might origin from renal tubules in DN (21). Thus, the causative factor of renal tubular injury in DN is supported by diabetic patients and experimental models, as well as credible pathogenetic mechanisms (22).

Intra-renal oxidative stress plays a critical role in the initiation and development of DN. There is considerable evidence that hyperglycemia causes oxidative stress through the increased generation of ROS, which plays a key role in DN (23). The increased MDA, the main aldehyde product of lipid peroxidation (24), and the decreased SOD, CAT, and GSH-Px were also noted in kidneys of diabetic animals (25, 26). For the sensitivity of renal tubules to oxidative stress and lipid peroxidation (27), ferroptosis often occurs in tubules during the development of renal diseases (28, 29). However, the researches of relation between ferroptosis and DN have been few in number. A recently published study reported that ferroptosis involved in renal tubular cell death in diabetic nephropathy (30). While clearly of great importance, there were still some limitations in that study. In vitro part of the research, renal tubular cells were not cultured under high glucose condition; in vivo study, the injury of renal tubules was not detected, and diabetic models were not treated with ferroptosis inhibitor to verify the role of ferroptosis in DN. Thus, it is necessary to further investigate whether ferroptosis enhanced renal tubular injury caused by diabetes (30).

It has been demonstrated that ferroptosis plays a crucial role in renal ischemia injury (1). DN is one of the diabetic microvascular complications. Renal ischemia has been considered as one of the major causes of DN (31). The high energy requirements and dependence on aerobic metabolism render renal tubules especially susceptible to hypoxia (17). Chronic hypoxia due to renal ischemia induces the increase of HIF-1α in renal tubules of diabetic models, with the elevated HO-1 level (32). Degradation of heme by the excessive HO-1 leads to iron overloading which causes oxidative stress and lipid peroxidation. Recent studies have documented the great effects of iron accumulation in kidneys on the progression of DN (33, 34). These studies suggested that the process of ferroptosis might affect the development of DN, diabetic renal tubular injury in particular, through HIF-1α/HO-1 pathway. However, few studies have explored it.

Furthermore, diabetic renal tubular injury contributes to renal fibrosis (35), and ferroptosis is regarded as the cause of fibrosis (36, 37). It has been proven that tubular epithelial cells under high glucose condition exhibited higher activation of pro-inflammatory and pro-fibrotic signal pathways, which led to progressive fibrosis (38). Given that the degree of renal tubular dysfunction associates well with the extent of renal fibrosis, diabetic tubular injury might be recognized as the reason of renal fibrosis (39). Moreover, our previous study showed that endothelial-specific prolyl hydroxylase domain protein-2 knockout (PHD2ECKO) mice, with the upregulated expression of HIF-α due to the deficient PHD2 which degrades HIF, presented significant renal fibrosis (40). However, whether ferroptosis-induced-renal fibrosis is regulated by HIF-1α/HO-1 pathway has been unclear.

Therefore, in this study, we aimed to investigate whether ferroptosis involved in tubular injury and fibrosis through HIF-1α/HO-1 pathway in kidneys of diabetic mouse models.

## Materials and Methods

The animal experiments were approved by the Animal Ethics Committee of Beijing Chao-Yang Hospital, Capital Medical University and were performed in accordance with animal care guidelines of Beijing Chao-Yang Hospital, Capital Medical University.

### Experimental Animal Models and Treatment

Eight-week-old male C57BLKs/J db/m and db/db mice were purchased from Nanjing Biomedical Research Institute of Nanjing University, Nanjing, China. Mice were divided into 3 groups (n = 9 for each group): (1) db/m group, (2) db/db group, and (3) db/db+Fer1 group. Db/db mice were given daily intraperitoneal injections of either 0.1% DMSO (diluted in 0.9% NaCl with 20% SBE-β-CD) for db/db group or 1 mg/kg Ferrostatin-1 (MCE, NJ, USA) for db/db+Fer1 group for 10 weeks starting at 10 weeks of age. Ferrostatin-1 was dissolved in DMSO first, and was diluted in 0.9% NaCl with 20% SBE-β-CD. The final concentrations of Ferrostatin-1 and DMSO were 0.2 mg/ml and 0.1%, respectively.

The mice were housed in clear plastic cages (n = 3/cage) at 22°C on a 12:12 h light-dark cycle (lights on 08:00–20:00 h), with free access to standard rodent chow and tap water. After 10-week administration, all mice were placed in metabolic cages separately to take 24 h urine. After 10 weeks, all mice were anesthetized by intraperitoneal injection of a mixture of Rompun 10 mg/kg (Bayer Korea, Ansan, Gyeonggi-Do, Korea) and Zoletil 30 mg/kg (Virbac, Carros, France). The kidneys were rapidly dissected for subsequent analyses. Blood was collected from the left ventricle and centrifuged, stored at -80°C.

### Measurements of Blood and Urinary Parameters

Fasting blood glucose concentration was measured using HemoCue B-Glucose kit (HemoCue AB, Angelholm, Sweden). Fasting insulin concentration was measured using radioimmunoassay kit (Linco Reasearch, St Charles, MO, USA). Serum and urine creatinine values were measured using HPLC (Beckman Instruments, Fullerton, CA, USA). Urinary albumin value was measured by an immunoassay (Bayer, Elkhart, IN, USA). Urinary albumin-to-creatinine ratio (UACR) was calculated as urine albumin/urine creatinine (μg/mg). Urinary kidney injury molecular-1 (KIM-1) and neutrophil gelatinase-associated lipocalin (NGAL) concentrations of mice were measured with ELISA (R&D systems, MN, USA). Serum iron ion, ferritin, and transferrin were determined with ELISA (Lai Er Bio-Tech, Hefei, China). All assays were performed according to the manufacturer’s protocol.

### Light Microscopic Study

The renal tissues fixed in neutral-buffered 10% formalin solution (SF93-20; Fisher Scientific, Pittsburgh, PA, USA). Paraffin sections were prepared in 8 µm. Apoptosis in kidneys was detected by TUNEL (Boster, Wuhan, China). Hexamine silver staining was performed to detect the injury of renal tubules. Masson’s trichrome staining and Sirius red staining were performed to measure the degree of renal fibrosis. Then, slices were washed with distilled water, and were dipped in Lillie staining solution (Solarbio Life Sciences, Beijing, China) for 30 min. Subsequently, slices were immersed in nucleus staining solution (Solarbio Life Sciences, Beijing, China) for 5 min, after washed by distilled water. Finally, dehydrated slices were used for measuring iron content after washed with distilled water again. The renal samples were also embedded in frozen optimal cutting temperature compound (4585; Fisher Health Care, Houston, TX, USA). Frozen sections were also prepared in 8 µm. ROS (frozen sections) was measured by dihydroethidium staining in fresh frozen sections. All analyses were performed by image-analysis software (Image J, NIH, Bethesda, MD, USA).

### Western Blot Analyses

Mouse renal tissues were collected and homogenized in lysis buffer. The homogenates were centrifuged at 16,000×g at 4°C for 15 min. A bicinchoninic acid protein assay kit (Pierce Co, Rockford, IL, USA) was used to analyze the protein concentrations. Equal amounts (20 µg) of the protein were separated by 10% sodium dodecyl sulfate polyacrylamide gel electrophoresis geland transferred to a polyvinylidene difluoride membrane. The membranes were blocked with 5% nonfat dry milk in Tris-buffered saline and incubated with the following primary antibodies overnight: cleaved caspase-3 (1:1000; Abcam, Cambridge, MA, USA), KIM-1 (1:1,000; Abcam, Cambridge, MA, USA), NGAL (1:1,000; Abcam, Cambridge, MA, USA), collagen I (1:1,000; Abcam, Cambridge, MA, USA), HIF-1α (1:1,000; Novus Bio, Littleton, CO, USA), HO-1 (1:1,000; BD transduction, San Jose, CA, USA), ferritin heavy chain (1:1000; Abcam, Cambridge, MA, USA), gp91 phox (1:1,000; BD transduction, San Jose, CA, USA), GPX4 (1:1,000; Abcam, Cambridge, MA, USA), and β-actin (1:1,000; Cell Signaling, Danvers, MA, USA). After washed, the membranes were incubated for 2 h with a secondary antibody coupled to horseradish peroxidase (1:5,000; Santa Cruz, CA, USA). Densitometric analyses were carried out with image acquisition and analysis software (Bio-Rad).

### SAssessment of Oxidative Stress Parameters in Mouse Renal Tissues

MDA was measured by thiobarbituric acid method, SOD was measured by xanthine oxidase method, GSH-Px was measured by NADPH method, and CAT was determined by coloration method in the renal tissue sample homogenates using commercial kits (Beyotime Institute of Biotechnology, Shanghai, China), according to the manufacturer's protocols.

### Statistical Analyses

All analyses were performed using Statistical Package for Social Sciences version 20.0 (SPSS, Inc., Chicago, IL, USA). Data are expressed as means ± S.E.M. The significance of differences in the means of corresponding values among groups was determined by using the one-way ANOVA. The significance of differences between two values was determined using LSD test. In all statistical tests, all tests were two-sided, and *P* values <0.05 were considered significant.

## Results

### Assessment of Physical and Biochemical Characteristics

**Figure 1:**
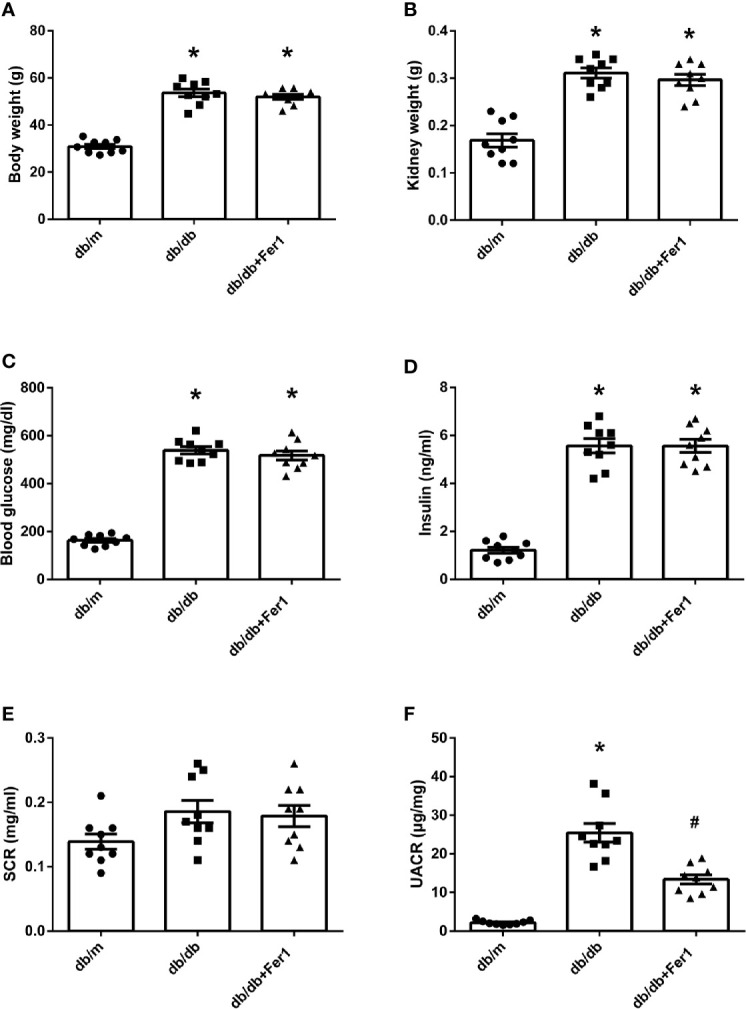
Physical and biochemical characteristics in db/m, db/db, and db/db+Fer1 groups. **(A)**. Body weight. **(B)**. Kidney weight. **(C)**. Blood glucose. **(D)**. Insulin. **(E)**. Serum creatinine (SCR). **(F)**. Urinary albumin-to-creatinine ratio (UACR). Male mice, n = 9/group. *P < 0.05, vs db/m group; ^#^ *P* < 0.05, vs db/db group. Db/m, db/m mice; db/db, db/db mice without Ferrostatin-1 treatment; db/db+Fer1, db/db mice with Ferrostatin-1 treatment. Data are means ± S.E.M.

As shown in **Figure 1**, body weight, kidney weight, blood glucose, and insulin were significantly higher for db/db mice than db/m mice, and there was no difference in body weight, kidney weight, blood glucose, and insulin in db/db mice between with and without Ferrostatin-1 treatment (**Figures 1A–D**). All mice in three groups were similar in serum creatinine (SCR) (**Figure 1E**). In addition, db/db mice presented higher UACR than db/m mice, and db/db+Fer1 group had the significantly decreased level of UACR compared with db/db group (**Figure 1F**), suggesting that ferroptosis was involved in DN.

### Assessment of Renal Tubular Injury

**Figure 2:**
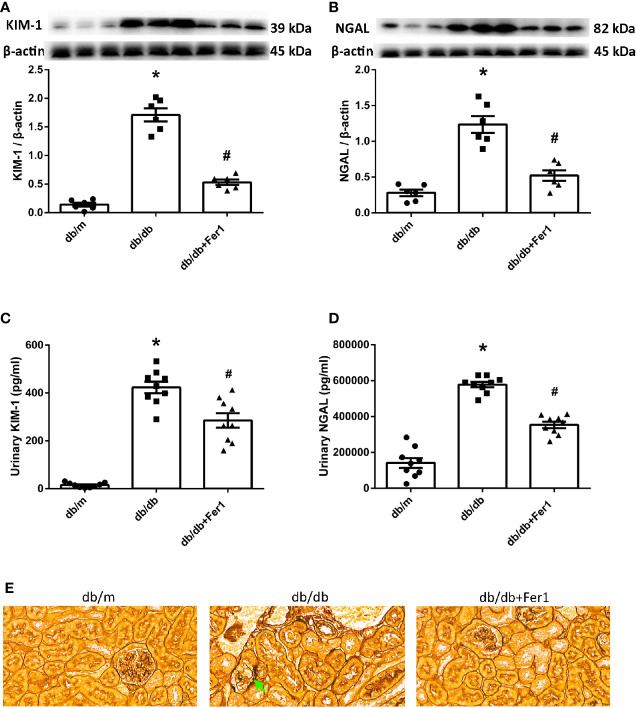
Renal tubular injury in db/m, db/db, and db/db+Fer1 groups. **(A, B)**. Representative photographs and quantification of kidney injury molecular-1 (KIM-1) **(A)** and neutrophil gelatinase-associated lipocalin (NGAL) **(B)** in mouse kidneys measured by western blot. **(C, D)**. Quantification of urinary KIM-1 **(C)** and NGAL **(D)** levels measured by ELISA. **(E)** Representative photographs of mouse kidneys by hexamine silver staining staining. Male mice, n = 6-9/group. *P < 0.05, vs db/m group; ^#^ *P* < 0.05, vs db/db group. Db/m, db/m mice; db/db, db/db mice without Ferrostatin-1 treatment; db/db+Fer1, db/db mice with Ferrostatin-1 treatment. Data are means ± S.E.M.

Since the renal tubules are vulnerable to metabolic disorders and ischemia, defects in tubules might be the primary cause of albuminuria in DN (17). To determine if there was ferroptosis-related renal tubular injury in diabetic mice, we detected the levels of KIM-1 and NGAL, the markers of renal tubular damage. Western blot showed that diabetes promoted the expression of KIM-1 and NGAL in the mouse kidneys, but ferroptosis inhibitor Ferrostatin-1 reduced the expression of KIM-1 and NGAL in db/db mouse kidneys (**Figures 2A, B**). Consistent with these changes, urinary KIM-1 and NGAL were increased in db/db group compared with db/m group, and were decreased after Ferrostatin-1 treatment in db/db mice (**Figures 2C, D**). Furthermore, hexamine silver staining showed that diabetes led to fold and fault of renal tubular basement membranes (**Figure 2E**). However, Ferrostatin-1 treatment improved the injury of renal tubular basement membranes in db/db mice. These results indicated that ferroptosis enhanced diabetic renal tubular injury.

### Assessment of Renal Fibrosis

**Figure 3:**
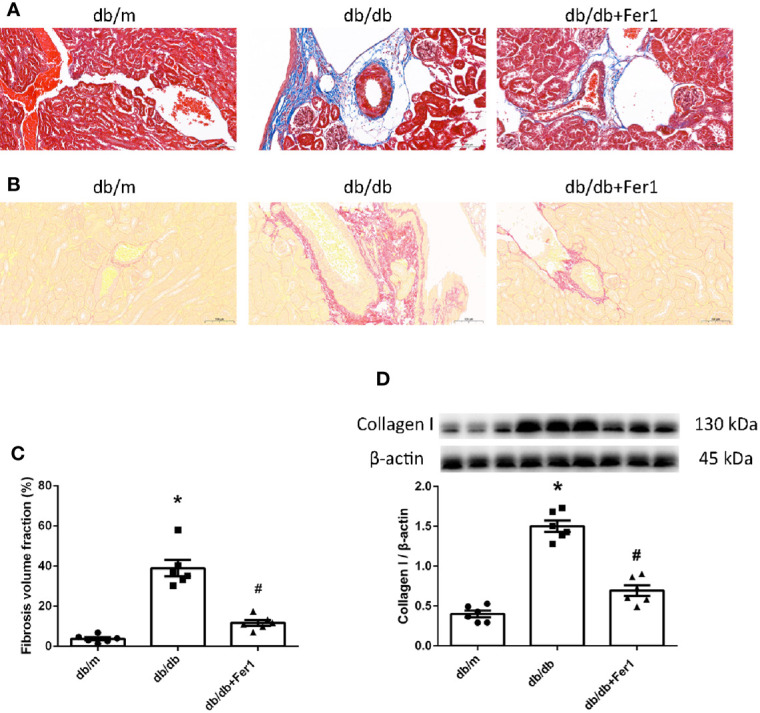
Renal fibrosis in db/m, db/db, and db/db+Fer1 groups. **(A–C)**. Representative photographs and quantification of renal fibrosis by Masson’s staining (blue) **(A)** and Sirius red staining (red) **(B)** (six sections per mouse were analyzed). **(D)**. Representative photographs and quantification of collagen I in mouse kidneys measured by western blot. Male mice, n = 6/group. *P < 0.05, vs db/m group; ^#^ *P* < 0.05, vs db/db group. Db/m, db/m mice; db/db, db/db mice without Ferrostatin-1 treatment; db/db+Fer1, db/db mice with Ferrostatin-1 treatment. Data are means ± S.E.M.

The previous researches have provided a strong evidence of ferroptosis to accelerate fibrosis (36, 37). Thus, we examined renal fibrosis. As shown in **Figure 3**, Masson’s staining and Sirius red staining showed that diabetes significantly enhanced mouse renal fibrosis, and Ferrostatin-1 treatment reduced renal fibrosis in diabetic mice (**Figures 3A–C**). Western blot analysis further showed that diabetes promoted the expression of fibrosis associated protein-collagen I in mouse kidneys, and Ferrostatin-1 treatment depressed the expression of collagen I in kidneys of db/db mice (**Figure 3D**). These results indicated that ferroptosis accelerated renal fibrosis in diabetic mice.

### Assessment of HIF-1α and HO-1 in Mouse Kidneys

**Figure 4:**
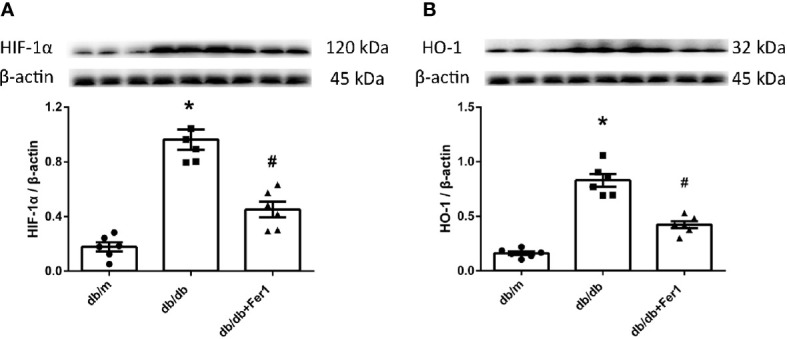
The expression of hypoxia-inducible factor (HIF) -1α and heme oxygenase (HO) -1 in mouse renal tissues. **(A, B)**. Representative photographs and quantification of HIF-1α **(A)** and HO -1 **(B)** in mouse kidneys measured by western blot. Male mice, n = 6/group. *P < 0.05, vs db/m group; ^#^ *P* < 0.05, vs db/db group. Db/m, db/m mice; db/db, db/db mice without Ferrostatin-1 treatment; db/db+Fer1, db/db mice with Ferrostatin-1 treatment. Data are means ± S.E.M.

Subsequently, we measured the expression of HIF-1α and HO-1 in mouse kidneys, since HO-1 has been suggested to act as a critical role in ferroptosis (10), and HIF-1α adjusts the expression of HO-1 (15). As exhibited in **Figure 4**, western blot showed that the levels of HIF-1α and HO-1 were increased in db/db mouse kidneys compared with db/m mouse kidneys, while Ferrostatin-1 treatment decreased the levels of HIF-1α and HO-1 in kidneys of db/db mice (**Figures 4A, B**). These results suggested that HIF-1α/HO-1 pathway might involve in ferroptosis-induced DN.

### Assessment of Iron Content

**Figure 5:**
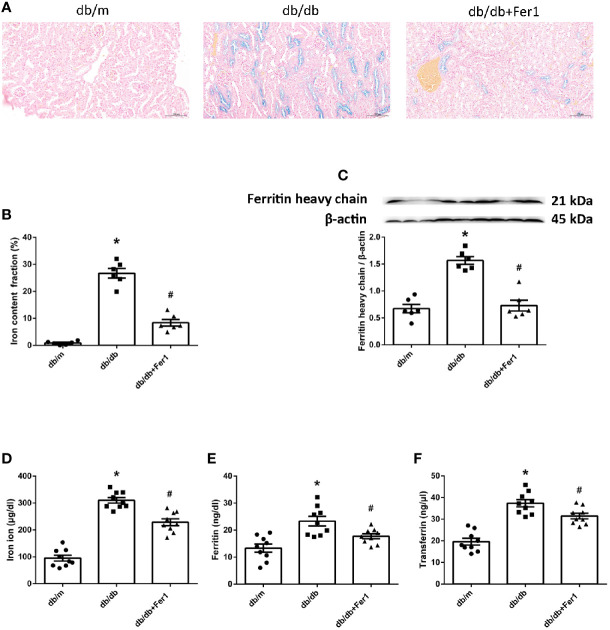
Iron content in db/m, db/db, and db/db+Fer1 groups. **(A, B)**. Representative photographs and quantification of iron content (blue) in kidneys by Lillie staining (six sections per mouse were analyzed). **(C)**. Representative photographs and quantification of ferritin heavy chain in mouse kidneys measured by western blot. **(D–F)**. Quantification of serum iron ion **(D)**, ferritin **(E)**, and transferrin **(F)** measured by ELISA. Male mice, n = 6–9/group. *P < 0.05, vs db/m group; ^#^ *P* < 0.05, vs db/db group. Db/m, db/m mice; db/db, db/db mice without Ferrostatin-1 treatment; db/db+Fer1, db/db mice with Ferrostatin-1 treatment. Data are means ± S.E.M.

Iron overloading is a risk factor for many disorders, because iron regulates considerable enzymes which are involved in lipid peroxidation and oxidative stress (2, 3). Ferroptosis is a form of regulated cell death resulting from iron overloading (1). Therefore, we measured the iron content in mouse kidneys and blood. As shown in **Figure 5**, Lillie staining showed that diabetes increased the iron content in mouse renal tubules, which was relieved in diabetic mouse renal tubules after Ferrostatin-1 treatment (**Figures 5A, B**). Western blot showed that the expression of ferritin heavy chain was increased in db/db mouse kidneys compared with db/m mouse kidneys, but Ferrostatin-1 reduced the expression of ferritin heavy chain in kidneys of db/db mice (**Figure 5C**). Additionally, db/db group had higher serum iron ion, ferritin, and transferrin in comparison to db/m group, but Ferrostatin-1 treatment inhibited these parameters in db/db mice (**Figures 5D–F**). These results showed that diabetes contributed to iron overloading in mouse renal tubules, but ferroptosis inhibitor alleviated iron overloading in renal tubules of db/db mice.

### Assessment of ROS Formation in Mouse Kidneys

**Figure 6:**
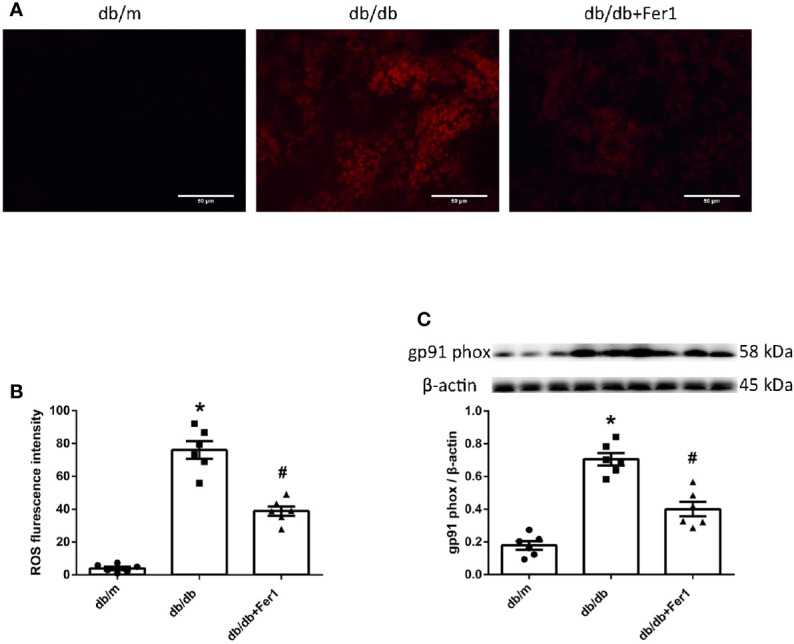
ROS formation in mouse renal tissues. **(A, B)**. Representative photographs and quantification of ROS formation (red) in mouse kidneys by dihydroethidium staining (six sections per mouse were analyzed). **(C)**. Representative photographs and quantification of gp91 phox in mouse kidneys measured by western blot. Male mice, n = 6/group. *P < 0.05, vs db/m group; ^#^ *P* < 0.05, vs db/db group. Db/m, db/m mice; db/db, db/db mice without Ferrostatin-1 treatment; db/db+Fer1, db/db mice with Ferrostatin-1 treatment. Data are means ± S.E.M.

Next, we detected ROS formation in mouse renal tissues. As presented in **Figure 6**, dihydroethidium staining showed that diabetes exacerbated ROS formation in mouse kidneys, while Ferrostatin-1 treatment depressed ROS formation in db/db mouse kidneys (**Figures 6A, B**). Moreover, diabetes resulted in a similar increased expression of NADPH oxidase subunit-gp91 phox in mouse kidneys, whereas Ferrostatin-1 treatment lessoned gp91 phox in db/db mouse kidneys (**Figure 6C**). These findings documented that diabetes promoted NADPH oxidase-derived ROS formation in mouse kidneys, which was suppressed by ferroptosis inhibitor Ferrostatin-1 treatment in diabetic mouse kidneys.

### Assessment of Lipid Peroxidation and GPX4 in Mouse Kidneys

**Figure 7:**
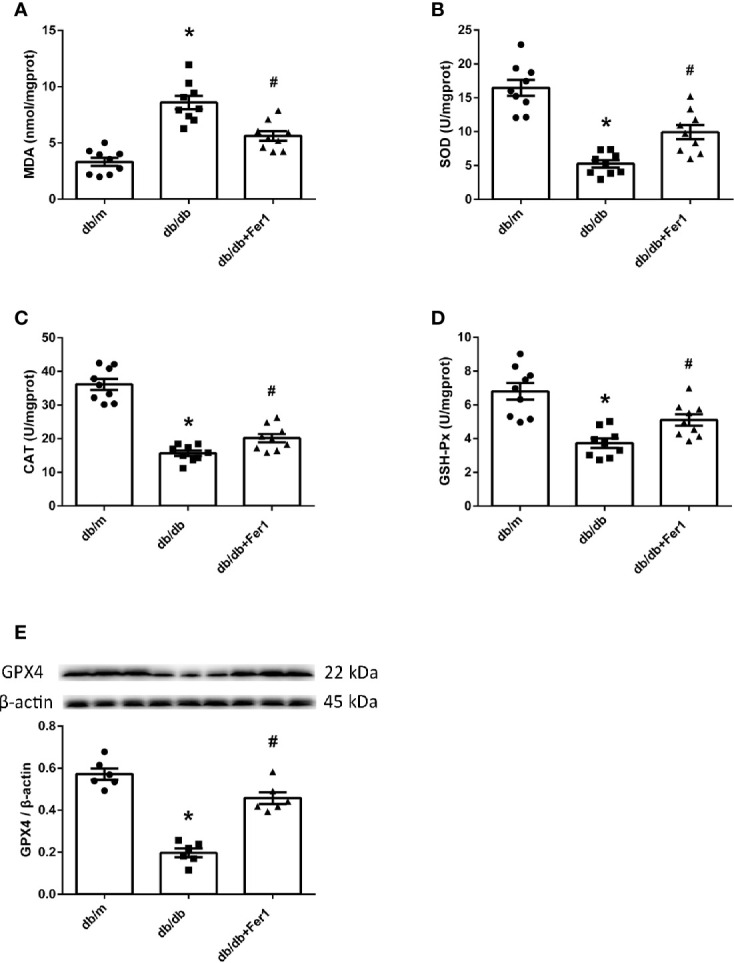
Lipid peroxidation and GPX4 in mouse renal tissues. **(A–D)**. Quantification of malondialdehyde (MDA) **(A)**, superoxide dismutase (SOD) **(B)**, catalase (CAT) **(C)** and glutathione peroxidases (GSH-Px) **(D)** in mouse kidneys. **(E)**. Representative photographs and quantification of glutathione peroxidase 4 (GPX4) in mouse kidneys measured by western blot. Male mice, n = 6–9/group. *P < 0.05, vs db/m group; ^#^ *P* < 0.05, vs db/db group. Db/m, db/m mice; db/db, db/db mice without Ferrostatin-1 treatment; db/db+Fer1, db/db mice with Ferrostatin-1 treatment. Data are means ± S.E.M.

Then, we assessed oxidative stress and lipid peroxidation in mouse renal tissues. As exhibited in **Figure 7**, compared with db/m group, the enhanced MDA and the reduced SOD, CAT and GSH-Px in mouse kidneys were noted in db/db group. In contrast, Ferrostatin-1 treatment resulted in the reduction on MDA and the enhancement on SOD, CAT and GSH-Px in kidneys of db/db mice (**Figures 7A–D**). Importantly, we measured the expression of GPX4 in mouse kidneys. Western blot showed that diabetes caused the decreased expression of GPX4 in mouse kidneys, but Ferrostatin-1 treatment increased the expression of GPX4 in kidneys of db/db mice (**Figure 7E**). These findings further indicated that lipid peroxidation induced-ferroptosis was involved in DN, which was improved by ferroptosis inhibitor Ferrostatin-1.

### Assessment of Renal Apoptosis

**Figure 8:**
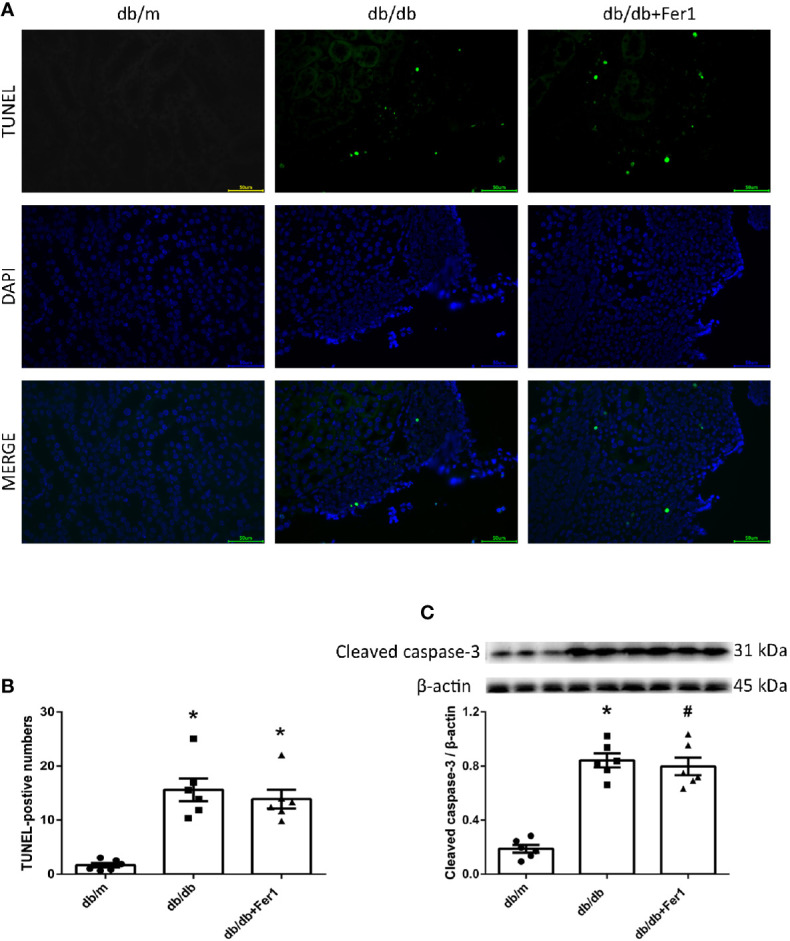
Renal apoptosis in db/m, db/db, and db/db+Fer1 groups. **(A, B)**. Representative photographs and quantification of renal apoptosis by TUNEL assay (six sections per mouse were analyzed). **(C)**. Representative photographs and quantification of cleaved caspase-3 in mouse kidneys measured by western blot. Male mice, n = 6/group. *P < 0.05, vs db/m group; ^#^ *P* < 0.05, vs db/db group. Db/m, db/m mice; db/db, db/db mice without Ferrostatin-1 treatment; db/db+Fer1, db/db mice with Ferrostatin-1 treatment. Data are means ± S.E.M.

Apoptosis has been found to play a role in renal injury and fibrosis induced by diabetes (41). Therefore, we detected mouse renal apoptosis. As shown in **Figure 8**, TUNEL assay showed that diabetes significantly promoted mouse renal apoptosis, but Ferrostatin-1 treatment did not relieve renal apoptosis in diabetic mice (**Figures 8A, B**). Moreover, western blot analysis showed that diabetes enhanced the expression of apoptosis associated protein-cleaved caspase-3 in mouse kidneys, while Ferrostatin-1 treatment did not suppress the expression of cleaved caspase-3 in kidneys of diabetic mice (**Figure 8C**). These results showed that diabetes accelerated apoptosis in mouse kidneys, but ferroptosis inhibitor Ferrostatin-1 treatment could not improve apoptosis in mouse kidneys.

## Discussion

In present study, we found that diabetes increased UACR level in mice, and diabetic state enhanced tubular injury, promoted fibrosis, elevated the levels of HIF-1α and HO-1, accelerated tubular iron overloading, and exacerbated ROS formation, oxidative stress, and lipid peroxidation in mouse kidneys. On the contrary, ferroptosis inhibitor Ferrostatin-1 decreased the level of UACR in diabetic mice, and Ferrostatin-1 improved tubular injury, reduced fibrosis, repressed the expression of HIF-1α and HO-1, suppressed tubular iron overloading, and inhibited ROS formation, oxidative stress, and lipid peroxidation in diabetic mouse kidneys.

Ferroptosis is a kind of regulated cell death characterized by iron-dependent accumulation of lipid peroxides. Ferroptosis associates well with renal ischemia injury (1) that is one of the major causes of DN (31). Our present research showed that diabetic mice presented higher UACR than control mice, but ferroptosis inhibitor Ferrostatin-1 reduced the level of UACR in diabetic mice, suggesting that ferroptosis was involved in the pathogenesis of DN, and inhibition of ferroptosis can protect against DN.

Initially, the underlying mechanism of albuminuria in DN has been attributed to the increased glomerular leakage. While great importance, changes in glomeruli may not be the main determinant in the prognosis of DN. As renal tubular injury participates in the development of DN, interest in the mechanism of DN has transferred to the renal tubules (35). As a result of emerging evidence supporting a role for ferroptosis in damaging renal tubules (28–30), we detected the damage of renal tubules in diabetic mice. In current study, diabetic mice presented higher KIM-1 and NGAL, the markers of renal tubular damage, both in kidneys and in urine, as compared to non-diabetic mice, with renal tubular basement membranes folding and faulting by hexamine silver staining. However, these changes about renal tubular injury were ameliorated in diabetic mice after ferroptosis inhibitor Ferrostatin-1 treatment. These results provided a strong evidence of ferroptosis to participate in renal tubular injury in diabetic mice.

Ferroptosis has been considered as a trigger of many diseases, and it is also associated with fibrosis (36, 37). Because renal tubular dysfunction is correlated with the extent of renal fibrosis, tubular injury induced by diabetes is identified as the cause of renal fibrosis (39). In current study, both Masson’s staining and Sirius red staining showed that there was a significant increase in the renal fibrosis area in diabetic mice as compared to non-diabetic mice. Consistent with the changes of renal fibrosis fractional area, the expression of fibrosis-associated protein-collagen I was significantly increased in diabetic mouse kidneys as compared to non-diabetic mouse kidneys. However, all changes of renal fibrosis were alleviated by ferroptosis inhibitor Ferrostatin-1 treatment in diabetic mice, which suggested that ferroptosis induced renal fibrosis in diabetic mice.

Previous studies have demonstrated intra-renal hypoxia in clinical patients and experimental models with diabetes (32, 42). The renal tubules are susceptible to the damage from metabolic disorders and hypoxia that are the pathogenesis of diabetes influencing the kidneys (39). The adaptation of hypoxia is mainly conferred through HIF-1α, which induces the expression of HO-1 (15) and regulates ferroptosis (10). HIF-1α and HO-1 have been demonstrated to serve the dual roles in multiple models of kidney injury, including DN. There have been some studies about the significant protective role of HIF-1α/HO-1 pathway (43). However, several recent studies have validated the promoted HIF-1α and HO-1 levels in kidneys of diabetic models (32). Furthermore, in contrast to the protective role in kidneys, our previous studies exhibited that the upregulated expression of HIF-α caused significant renal fibrosis (40), and that the increased HO-1 and iron overloading were presented in kidneys of hypertensive mice (44). Morover, genetic knockdown and pharmacological inhibition of HO-1 validated that activation of HO-1 triggers ferroptosis through iron overloading and subsequently excessive production of ROS and lipid peroxidation (11, 13). It is possible that these conflicting results are due to the poly-pharmacotherapy or other confounding variables of the experimental models, such as species, strains, gender, age and the diseases of models. These discrepancies might also be caused by differences in assays used in different researches. In current study, the increased levels of HIF-1α and HO-1 were observed in diabetic mouse kidneys compared with non-diabetic mouse kidneys. By contrast, ferroptosis inhibitor Ferrostatin-1 treatment reduced the levels of HIF-1α and HO-1 in kidneys of diabetic mice. These findings suggested that ferroptosis might enhanced DN through HIF-1α/HO-1 pathway.

The increased HO-1 contributes to more iron accumulation by accelerating degradation of heme. Iron is essential for cell survival. Iron overloading is a known risk factor for various disorders. Ferroptosis is a form of regulated cell death that is induced by iron overloading (1). Renal iron accumulation has been proposed to promote the progression of DN (33, 34). However, Ferrostatin-1 can form a complex compounded with iron (45). Previous studies have found that ferroptosis inhibitor Ferrostatin-1 alleviated atherosclerosis lesion and inhibited the iron accumulation in HFD-fed ApoE^-/-^ mice (46), protected against early brain injury and decreased the iron content in subarachnoid hemorrhage rats (47), and inhibited death of microglia and alleviated iron overloading induced by nitrogen-doped graphene quantum dots (48). In current research, Lillie staining showed that there was more iron accumulation in diabetic mouse renal tubules compared with non-diabetic mouse renal tubules, but iron overloading was relieved in renal tubules of diabetic mice after Ferrostatin-1 treatment. Ferritin and transferrin are regulated by HO-1, and the elevated HO-1 increases the levels of ferritin and transferrin (49). Western blot showed that ferritin heavy chain was promoted in diabetic mouse kidneys compared with non-diabetic mouse kidneys, while Ferrostatin-1 treatment decreased ferritin heavy chain in kidneys of diabetic mice. Additionally, diabetic mice had higher serum iron ion, ferritin, and transferrin than non-diabetic mice, but Ferrostatin-1 treatment inhibited these parameters in diabetic mice. These results showed that diabetes contributed to iron overloading in mouse renal tubules, but inhibition of ferroptosis alleviated iron accumulation in diabetic mouse renal tubules.

Ferroptosis occurs as a result of elevated ROS levels due to the increased intracellular iron concentration (1). Renal ROS generation in diabetes is predominantly mediated by NADPH oxidases (50). The excess of ROS caused by hyperglycemia plays a dominant role in DN (23). The current study showed that diabetes resulted in a significant elevation of ROS formation in mouse kidneys, and ferroptosis inhibitor Ferrostatin-1 induced the reduction of ROS formation in diabetic mouse kidneys. This was accompanied by a significant increased expression of NADPH oxidase subunit-gp91 phox in diabetic mouse kidneys compared with non-diabetic mouse kidneys, and ferroptosis inhibitor Ferrostatin-1 treatment suppressed gp91 phox in diabetic mouse kidneys. These findings indicated that diabetes accelerated NADPH oxidase-derived ROS formation in mouse kidneys, but inhibition of ferroptosis prevented ROS formation derived by NADPH oxidases in diabetic mouse kidneys.

Iron overloading results in the overproduction of ROS, which contributes to an excess of oxidative stress and lipid peroxidation owing to insufficient antioxidant pathways (6). MDA is the main aldehyde product of lipid peroxidation (24). The enzymatic antioxidants consist of SOD, CAT, GSH-Px, etc (6). Recent researches have verified the increased MDA and the decreased SOD, CAT and GSH-Px in kidneys of diabetic animals compared with kidneys of the controls (25, 26). GPX4 has been identified to specifically prevent ferroptosis by suppression of phospholipid peroxidation. Ferrostatin-1 is an antioxidant and generates the same anti-ferroptotic effect as GPX4 proven by previous researches (45). Our current findings presented the increased MDA and the decreased SOD, CAT, and GSH-Px in diabetic mouse kidneys compared to non-diabetic mouse kidneys. However, ferroptosis inhibitor Ferrostatin-1 treatment repressed MDA, and promoted SOD, CAT, and GSH-Px in diabetic mouse kidneys. Moreover, GPX4 was lower in diabetic mouse kidneys than non-diabetic mouse kidneys, whereas GPX4 was increased by ferroptosis inhibitor Ferrostatin-1 treatment in kidneys of diabetic mice. These data supported that lipid peroxidation induced-ferroptosis deteriorated DN in db/db mice.

In addition, previous researches have documented that apoptosis has involved in diabetic renal injury (41). Our current results showed that apoptosis damaged diabetic mouse kidneys, but inhibition of ferroptosis could not reverse the diabetic damages in mouse kidneys, indicating that Ferrostatin-1 protected against DN through another mechanism instead of apoptosis, and ferroptosis might play a critical role in pathogenesis of DN.

## Conclusions

In summary, diabetes led to the increased UACR in mice, and diabetes further resulted in a significant mouse renal tubular injury and mouse renal fibrosis, a promotion of HIF-1α and HO-1 levels in mouse kidneys, an elevation of iron accumulation in mouse renal tubules, and an increase of lipid peroxidation due to the enhanced ROS generation in kidneys of mice; however, inhibition of ferroptosis decreased the UACR in diabetic mice, and inhibition of ferroptosis alleviated renal tubular injury and renal fibrosis in diabetic mice, repressed the levels of HIF-1α and HO-1 in diabetic mouse kidneys, reduced iron accumulation in renal tubules of diabetic mice, and prevented lipid peroxidation by decreasing ROS generation in kidneys of diabetic mouse models. Our study indicated that the process of ferroptosis might aggravate albuminuria, damage renal tubules, and enhance renal fibrosis in diabetic models through HIF-1α/HO-1 pathway, which may contribute to the further study on the pathogenesis of DN and provide a therapeutic target for DN.

## Data Availability Statement

The raw data supporting the conclusions of this article will be made available by the authors, without undue reservation.

## Ethics Statement

The animal study was reviewed and approved by the Animal Ethics Committee of Beijing Chao-Yang Hospital, Capital Medical University.

## Author Contributions

XF: design, experimentation, statistics, article revision. SW: experimentation. ZS: experimentation. HD: experimentation. HY: experimentation. MH: experimentation. XG: design, experimentation, statistics, article revision. All authors contributed to the article and approved the submitted version.

## Funding

This work was supported by grants from the Chinese National Natural Science Foundation (No. 81700713) to XF.

## Conflict of Interest

The authors declare that the research was conducted in the absence of any commercial or financial relationships that could be construed as a potential conflict of interest.
